# Methylation factor MRPL15 identified as a potential biological target in Alzheimer’s disease

**DOI:** 10.18632/aging.202862

**Published:** 2021-05-19

**Authors:** Li Gao, Jianmei Li, Ming Yan, Maimaiti Aili

**Affiliations:** 1 Prescription Laboratory of Xinjiang Traditional Uyghur Medicine, Xinjiang Institute of Traditional Uighur Medicine, Urmuqi 830011, China

**Keywords:** Alzheimer’s disease, risk genes, MRPL15, immune inflammation, methylation

## Abstract

Alzheimer’s disease (AD) is the most common form of dementia. However, the molecular basis of the development and progression of AD is still unclear. To elucidate the molecular processes related to AD, we obtained the expression profiles and analyzed the differentially expressed genes (DEGs). The genes potentially involved in the AD process were identified by PPI network and STEM analysis. The molecular mechanisms related to the recognition of AD were determined by GSEA and enrichment analysis. The differences from immune cells in AD were calculated. The methylation factors were identified by methylation difference analysis. Among them, MRPL15 was identified as suitable for diagnosing AD. Its expression trend had been verified in GSE5281. Importantly, MRPL15 was also a methylation factor. In addition, macrophages and neutrophils were up-regulated in AD patients. This was consistent with previous immune inflammation responses identified as being involved in the development of AD. The results of the present study revealed the genetic changes and molecular mechanisms involved in the process of the development and deterioration of AD patients. The potential AD risk genes and potential biological targets were identified. It provided evidence that immune inflammation and immune cells play an important role in AD.

## INTRODUCTION

Alzheimer’s disease (AD) is a complex and diverse neurodegenerative disease, which is clinically characterized by a decline in cognitive abilities and behavioral disorders [1, 2]. AD is the most common cause of dementia. The risk of AD increases with age, and the incidence rate is also increasing in the USA due to its aging population [3]. There are about 50 million people suffering from dementia in the world and Alzheimer’s disease accounts for 60-70% of cases [4]. It has been predicted that the incidence rate of AD will triple to over 15 million people by 2050, with an annual cost of over $ 700 billion [5]. Currently, there is a great need for effective preventative and curative treatments to either prevent, slow the progression or preferably cure AD.

Biomarkers are expected to promote the development of more effective drugs for treating AD and establish a more personalized medical treatment method. AD is defined by a combination of amyloid and τ proteins, accompanied by degeneration of neurons and their synapses, glial activation and neuroinflammation [6]. Synaptic dysfunction and loss are the events in early-onset AD [7]. The series of pathophysiological events of late-onset AD are not yet fully understood [8]. Microglia and astrocytes are the two main types of glial cells in the pathogenesis of AD. Inflammation is considered to be a factor in the pathogenesis of AD [9]. Microglia are immune effector cells of the central nervous system *in vivo*, and they play an important role in the dynamic balance of the brain and in immune response [10]. Astrocytes are the most abundant type of glial cells in the central nervous system. They play an important role in homeostasis, synapsis, signal transmission and synaptic plasticity, and in providing nutritional and metabolic support to neurons [11]. Brain insulin resistance caused by insulin-related disorders, is an additional pathological mechanism of AD [12]. AD is a multifactor disease involving genome, epigenome, and environmental factors [13]. Next generation molecular and high flux technologies are expected to elucidate the mechanism and network behind the complexity of AD. Epigenetic processes play a vital role in the central nervous system, especially DNA methylation modification [14]. The methylation of genes changes significantly with an increase in the age of people [15].

The accuracy of clinically diagnosing AD is generally low because of the absence of suitable biomarkers. A comprehensive, holistic and systematic analysis method is needed to describe and diagnose a complex multifactor disease. In the present study, we screened for and identified the genes of patients relevant to the different developmental stages of AD. The results of the present study improved the diagnosis and treatment targets of AD. In addition, it deepened our understanding of the developmental mechanism of AD.

## RESULTS

### Differentially expressed genes in the development of Alzheimer’s disease

**Figure 1:**
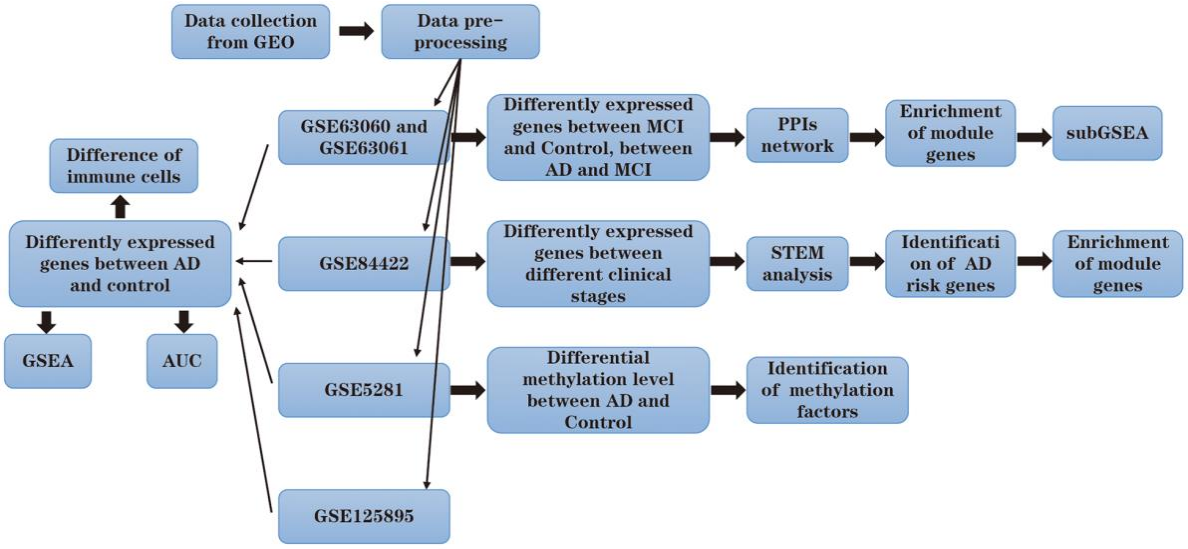
Flow chart.

**Figure 2:**
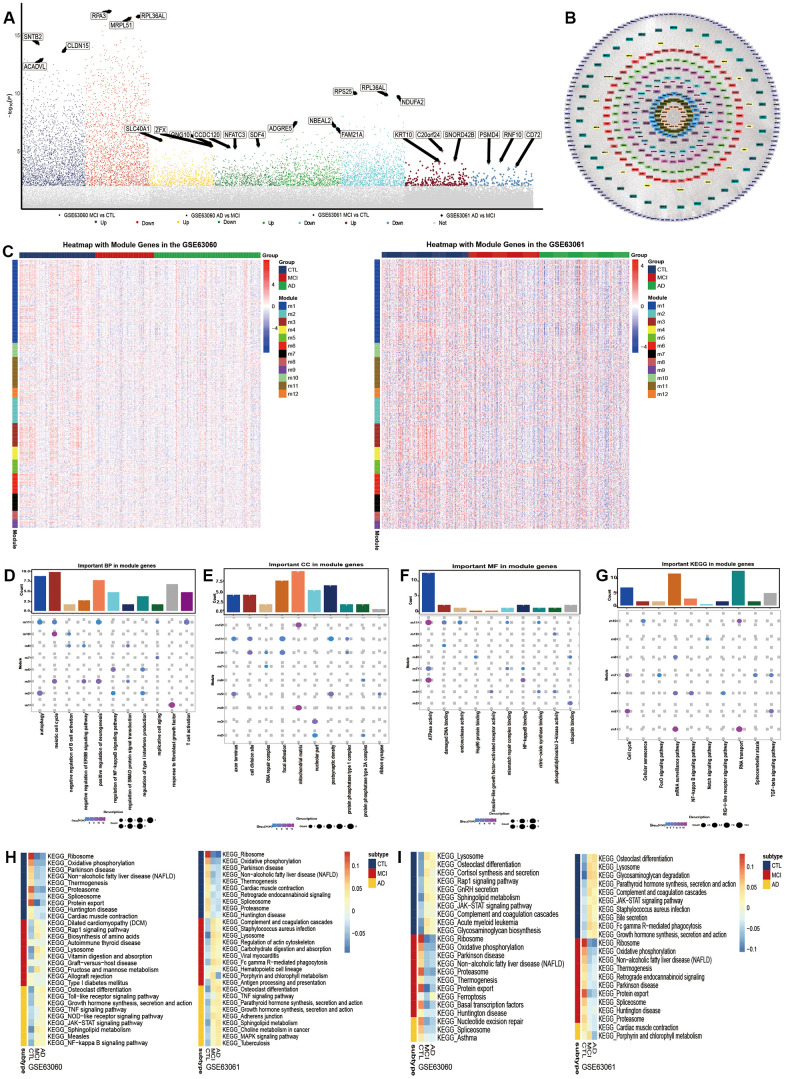
**Molecular mechanism in the development of Alzheimer’s disease.** (**A**) Differentially expressed genes between Alzheimer’s disease (AD) and mild cognitive impairment, and between mild cognitive impairment and the control. (**B**) The union of the two groups of the differentially expressed genes constitutes the PPI network. Each color represents a different module. (**C**) The expression heatmap of module genes in GSE63060 and GSE63061. The biological functions (**D**), cellular components (**E**) and molecular functions (**F**) of the modular genes. (**G**) The KEGG pathway of module genes. From control to mild cognitive impairment and then to AD, the signal pathway was continuously up-regulated (**H**) or down-regulated (**I**).

The research flowchart of the study is shown in Figure 1. To identify the changes in gene expression during the development of AD, we compared the differences in the GSE63060 and GSE63061 datasets. Among them, 3221 genes were differentially expressed between people with MCI and the control group, and 382 genes were differentially expressed between patients with AD and MCI (Figure 2A).

To identify the key genes in the process from mild cognitive impairment to Alzheimer’s disease, we obtained a PPI network composed of 1901 DEGs. These network genes were clustered into 12 interacting modules (Figure 2B). The trend in the expression of the modular gene in the two groups of data was similar, but the difference in the GSE63061 data was more obvious (Figure 2C). The enrichment analysis showed that the module genes were involved in 2107 biological processes (BP), 366 cell components (CC) and 385 molecular functions (MF). The involvement was mainly in the biological functions related to the nerves, in aging, immunity and inflammation (Figure 2D–2F). The results of the KEGG showed that the module genes were mainly enriched in the cell cycle, immune inflammation related signal pathway (Figure 2G). Importantly, in both datasets the results of the subGSEA indicated that Osteoclast differentiation and the Toll- like receptor signaling pathway showed a continuous up-regulation from healthy people to patients with AD (Figure 2H). However, Spliceosomes and Proteasomes continued to decrease (Figure 2I). It is suggested that these signaling pathways can promote or inhibit the development of Alzheimer’s disease.

### Molecular changes in the progression of Alzheimer’s disease

**Figure 3:**
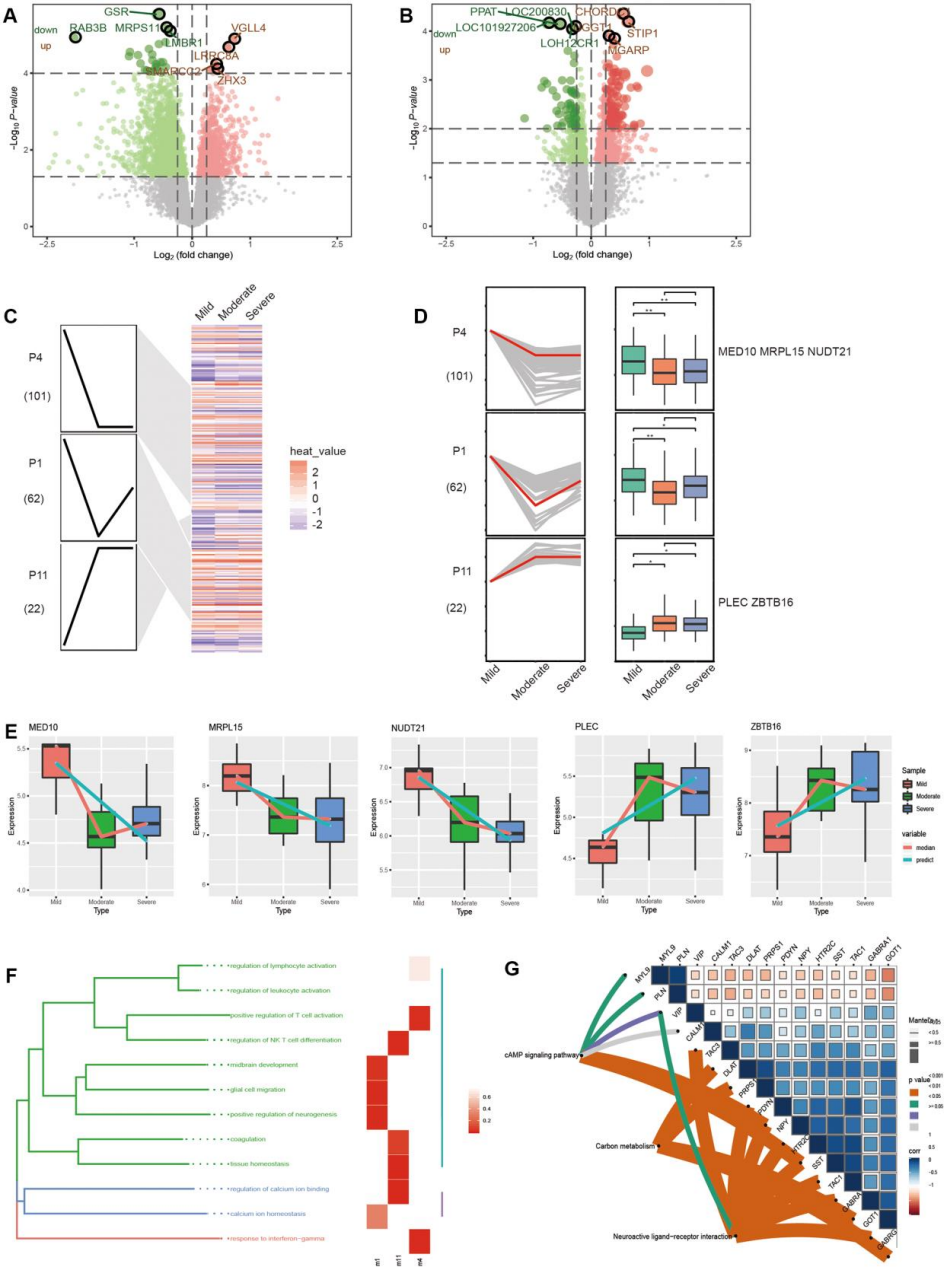
**Molecular changes in the progression of Alzheimer’s disease.** (**A**) The differentially expressed genes between moderate and mild AD patients. (**B**) Differentially expressed genes between severe and moderate AD patients. (**C**) Modules significantly up or down in the STEM results. (**D**) The trend in gene expression in significantly up-regulated or down-regulated modules. (**E**) The expression trend of potential risk genes in different stages of AD in patients. The enrichment results of persistent disorder genes include biological function (**F**) and the KEGG pathway (**G**).

There are different pathological stages in patients with AD. As AD worsens, the genes that continuously express maladjustment may be involved in this process. First, from the GSE84422 database, we obtained the DEGs between the patients with moderate and mild AD (Figure 3A), and between the patients with severe and moderate AD (Figure 3B). Then, by STEM analysis of these DEGs, we identified the genes that were continuously up-regulated or down-regulated from mild to severe AD (Figure 3C). Surprisingly, five genes (MED10, MRPL15, NUDT21, PLEC and ZBTB16) coincided with the PPI network genes (Figure 3D). We also determined the trend in the expression of these five genes in different modules of the STEM. The expression of the MED10, MRPL15 and NUDT21 genes were up-regulated, while the PLEC and ZBTB16 genes were down-regulated. We thought that these genes may be AD risk genes. Their specific expression trends in three different stages of AD were consistent with the up- and down-regulation of the STEM modules (Figure 3E). The enrichment analysis showed that the persistent disordered genes were mainly related to biological functions such as immune inflammation, neuroregulation, the cAMP signaling pathway, carbon metabolism, and neuroactive ligand−receptor interaction (Figure 3F, 3G).

### Molecular mechanism of Alzheimer’s disease

**Figure 4:**
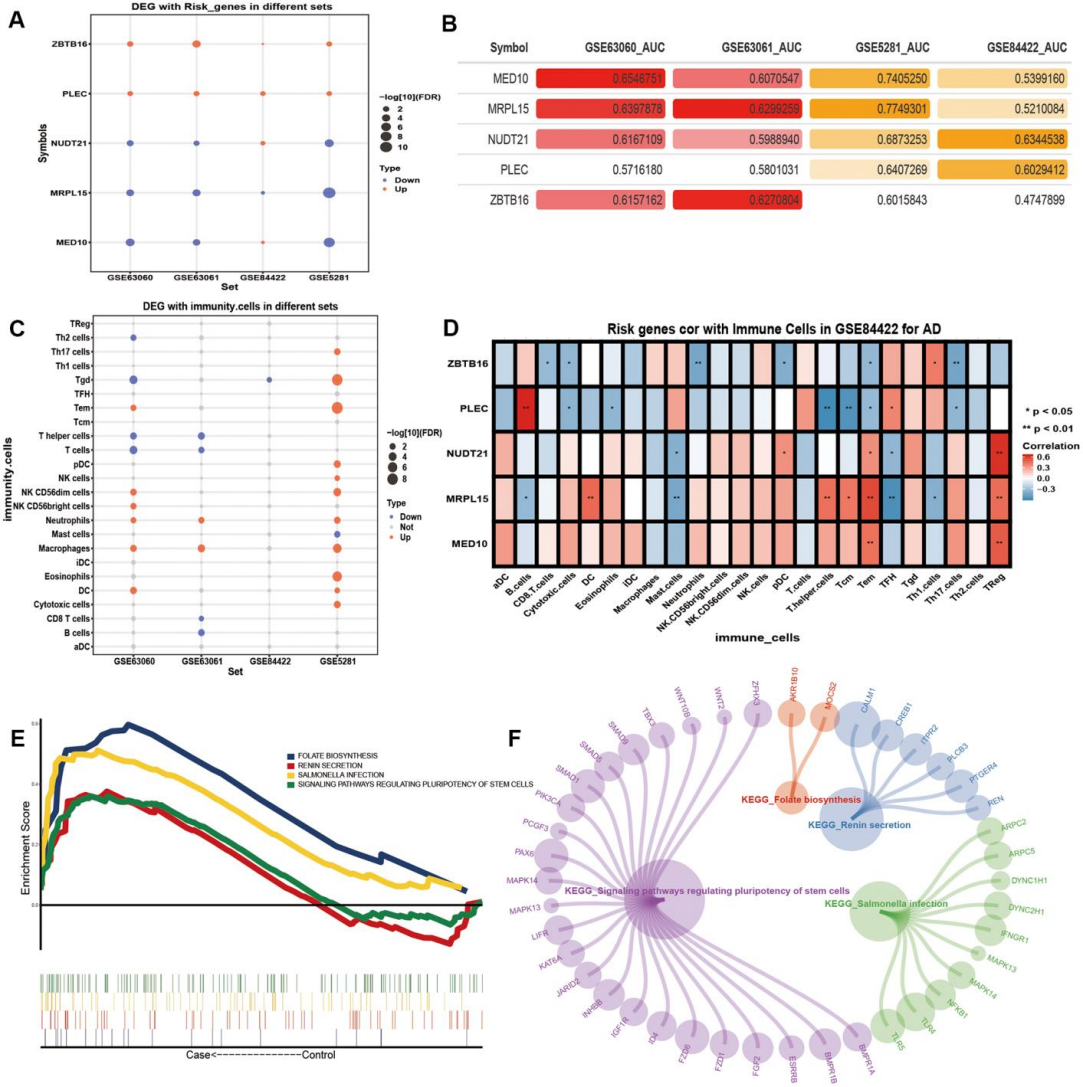
**The potential risk genes and immune changes in Alzheimer’s disease.** (**A**) Four data sets were used to verify the expression of the potential AD risk genes. (**B**) The AUC value of potential risk genes in AD patients. (**C**) The 24 differentially expressed immune cells. (**D**) Correlation between the potential AD risk genes and immune cells. (**E**) The GSEA results of gene expression in AD patients. (**F**) Gene expression of AD patients involved in the GSEA KEGG pathway.

Potential AD risk genes were identified by analyzing the differential expression of genes between the control samples and samples of people with AD (Figure 4A). In addition, potential AD risk genes, especially MED10 and MRPL15, were identified as being suitable for the clinical diagnosis of AD (Figure 4B). Among them, MRPL15 had the same expression direction and higher AUC value in the four groups of differentially expressed genes, indicating that it may serve as a biomarker of AD.

After the previous enrichment analysis, we found that immunity played an important role in the process of AD. Therefore, we identified the differences of the 24 kinds of immune cells in AD (Figure 4C). We found that Neutrophils and Macrophages were significantly up-regulated in all three datasets. We found that T cells and T helper cells were significantly down-regulated in the blood samples. We calculated the correlation between the potential AD risk genes and the immune cells. We found that MED10 and NUDT21 had the highest positive correlation with Treg cells. MRPL15 had the highest positive correlation with Tem cells. PLEC had the highest positive correlation with B cells. ZBTB16 had the highest positive correlation with Th1 cells (Figure 4D). The results of the GSEA showed that the expression of the five potential AD risk genes were mainly concentrated in the up-regulated salmonella infection, signaling pathways regulating pluripotency of stem cells (Figure 4E, 4F).

### Regulation of methylation of Alzheimer’s disease

**Figure 5:**
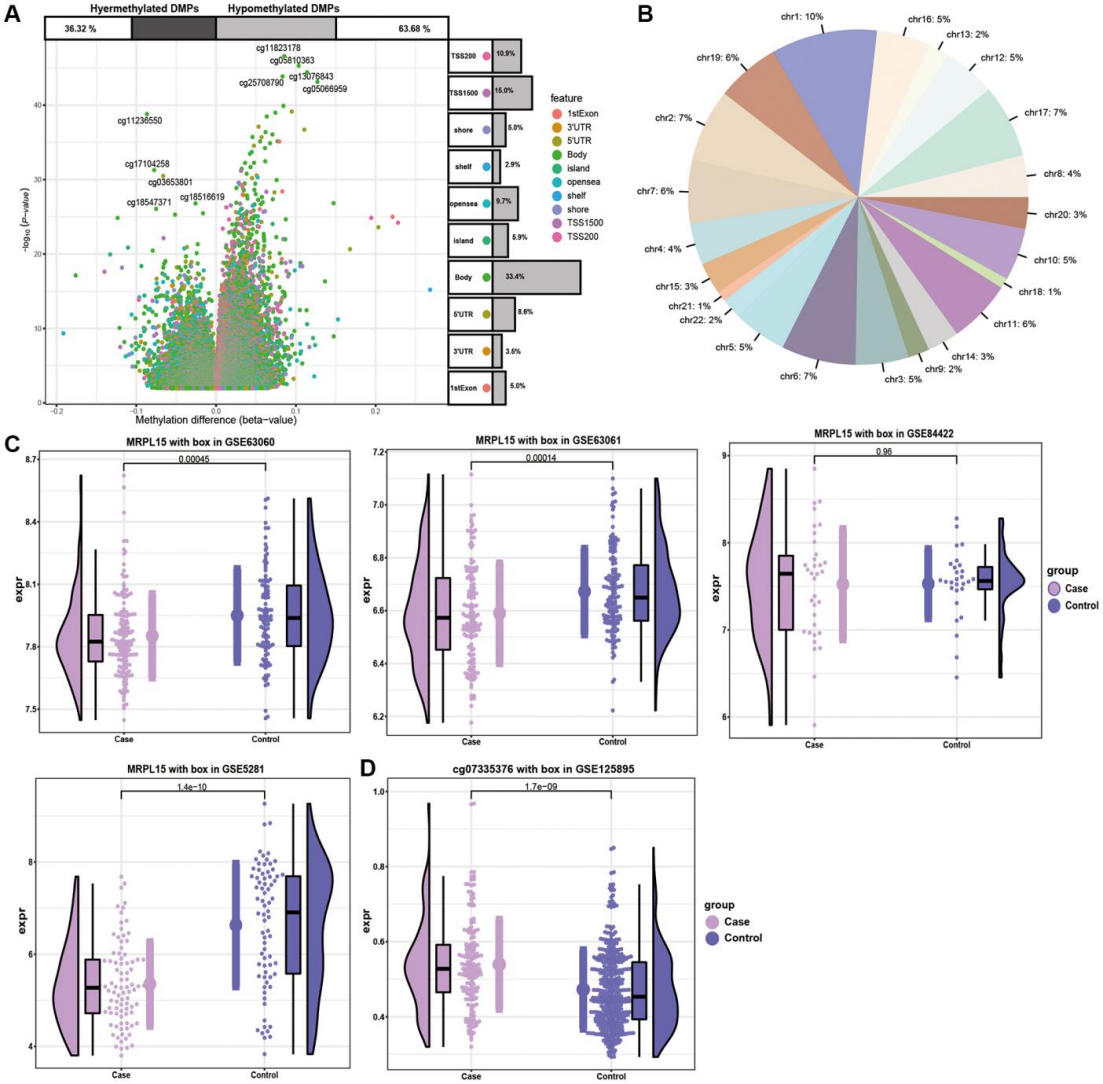
**The analysis of DMPs in AD cases and controls.** (**A**) Volcano plot of the top DMPs and position of methylation probes in relation to the gene. The percentages of hypermethylated and hypomethylated DMPs are displayed on top. (**B**) The proportion of methylation in different chromosomes. (**C**) Expression of MRPL15 in four datasets. (**D**) Methylation level of MRPL15.

DNA methylation plays an important role in AD by further affected DNA function by activating or inhibiting gene transcription activity [16]. By analyzing the methylation level of genes of AD patients, we identified a series of methylation sites (Figure 5A). In addition, the methylation level of chromosomes at different positions was also different (Figure 5B). The expression of MRPL15 was verified by four datasets (Figure 5C). The level of methylation of MRPL15 was opposite to its level of expression, which means that MRPL15 may be a methylation factor regulated by methylation (Figure 5D).

## DISCUSSION

We systematically studied the occurrence, development and deterioration of AD, as well as the related knowledge of the modification of DNA by methylation. There was evidence that hypomnesia in MCI patients was related to hippocampal atrophy and degeneration of the basal forebrain [17, 18]. MCI represents the transition state between normal aging and dementia disorders, especially AD [19]. Therefore, the DEGs among AD, MCI and control identified in this study may promote the development of AD. During the development of AD, Osteoclast differentiation and the Toll-like receptor signaling pathway were significantly activated, while Spliceosomes and Proteasomes were inhibited. Among them, the Toll-like receptor mediated important cellular immune responses when activated. New evidence suggested that Toll-like receptors were involved in the pathological process of AD [20]. The Proteasome system was an important intracellular protein degradation pathway, and ensures the balance of proteins in eukaryotic cells [21]. Proteasome dysfunction is a major cause or secondary consequence of many neurodegenerative diseases, including AD [22].

In addition, the spatiotemporal expression of genes in different stages of neuropathological diseases may indicate the diagnosis and treatment of the progression of a disease [23]. Five potential AD risk genes were identified through the study of gene persistent expression disorder at different stages of the development of AD in patients. Among them, the trend in the expression of MRPL15 was simultaneously verified by four datasets, and it had a good ability to be used to diagnose the occurrence of AD. Interestingly, MRPL15 was also methylated in AD. There was evidence that DNA methylation may be related to the potential risk of neurological diseases [24]. Therefore, MRPL15 was identified as a potential biomarker and therapeutic target. Consistent with our results, MRPL15 was down regulated in AD [25]. MRPL15 is a mitochondrial ribosomal protein necessary for protein synthesis. Mitochondrial dysfunction was found to be an important marker of AD [26, 27]. MRPL15 may also be related to the closure or disorder of the biological function of mitochondria and in the function of energy metabolism in schizophrenia [28].

In the process of DEG, we also found that the development and deterioration of AD were related to immune inflammation and neuroregulation and other biological effects. The studies of the genome wide association had shown that a large number of genes are related to the increased risk of AD. Many of these genes were expressed by immune cells, indicating that there was a major role of multicellular pathogenesis and neuroinflammation in the etiology of AD [29]. In fact, neuroinflammation was found to be an innate immune response of the nervous system, including microglia, astrocytes, cytokines and chemokines, which play a central role in the early stage of AD [30]. The pathophysiological mechanism of multifactor and polygenic AD was found to not only be limited to the tissue of neurons, but also related to brain immune response [31]. Our analysis showed that macrophages were significantly up-regulated in AD. Macrophages in the brain were thought to play a key role in the pathological immune response under load [32]. The number of Tem cells with the strongest correlation with MRPL15 was directly proportional to age [33].

The results of the present study enriched our understanding of the molecular mechanism of genetic changes in the process of AD development and deterioration. The genes found in the present study can be used as targets for further functional studies to further elucidate their diagnostic and therapeutic roles in AD.

## CONCLUSIONS

In the present study, we comprehensively analyzed the molecular changes in the occurrence and development of AD, and provided more biological connotations. We identified five potential AD related risk genes (MED10, MRPL15, NUDT21, PLEC and ZBTB16). Among them, MRPL15 was also the key gene modified by methylation. We also found that immunoinflammation and neuromodulation played an important role in the pathogenesis of AD. Our results showed that MRPL15 may be used as a molecular target of AD for further study.

## MATERIALS AND METHODS

### Data source and the identification of Differentially Expressed Genes (DEGs)

The following databases, GSE63060, GSE63061, GSE84422, GSE5281 and GSE125895, were downloaded from the Gene Expression Omnibus (GEO) database. The GSE63060 database includes blood samples from 104 controls, 80 people with mild cognitive impairment (MCI) and 145 AD patients. The GSE63061 database includes blood samples from 134 controls, 109 people with MCI and 139 AD patients. The GSE84422 database includes samples of brain tissue from 27 controls, and 7, 14 and 13 patients with mild, moderate and severe AD respectively. The GSE5281 database includes samples of brain tissue of 74 controls and 87 AD patients. The Illumina 450 K methylation array in the GSE125895 dataset includes the samples of brain tissue of 49 controls and 24 AD patients. Differentially expressed genes (DEGs), including up- and down-regulated genes, were identified using the R library ‘limma’. The DEGs were ultimately selected according to a false discovery rate, *P* < 0.05.

### Protein-Protein Interactions (PPIs) network

A protein-protein interaction (PPIs) network was established using the Search Tool for the Retrieval of Interacting Genes/Proteins (STRING) database. It was done by screening for scores greater than 900 and was based on the DEGs. The network was visualized using the software Cytoscape. The Molecular Complex Detection (MCODE) plugin was used to evaluate the biological importance of the constructed gene modules with an MCODE score greater than 6.

### Short Time-series Expression Miner (STEM)

The Java-based software STEM is specifically designed for the analysis of short time-series microarray gene expression data. Data of the normalized DEGs in each group was entered into the program, with all of the parameters set to their default values. The correlation coefficient was used to assign each gene to the closest profile. The p value derived from the STEM analysis was adjusted for multiple hypothesis testing, using a q value of less than 0.05. The profile boxes that were statistically significant were highlighted in different colors.

### Enrichment analysis and Gene Set Enrichment Analysis (GSEA)

To investigate the biological function altered in patients with AD, we performed functional annotations of the list of DEGs. The functional annotations included the Kyoto Encyclopedia of Genes and Genomes (KEGG) and the Gene Ontology (GO) (Biological Processes, the Molecular Function, and the Cellular Component) enrichment analysis. All of these functional annotations were performed with the R library ‘clusterProfiler’ using the pvalue Cutoff = 0.01 and qvalue Cutoff = 0.05. A GSEA was performed to elucidate the key pathways involved in the high vs low gene expression groups. A nominal *P* value < 0.05, a false discovery rate (FDR) < 0.05, and | NES | ≥ 1 were used to identify the significant pathways.

### Methylation analysis

The differentially methylated positions (DMPs) were estimated for the genes in the cAMP pathway in the control and AD patient groups from samples in the GSE125895 database. *P* < 0.05 was used as the cut-off standards to find DMGs.
